# Influence of Polygenic Risk on Height and BMI in Adults With a 22q11.2 Microdeletion

**DOI:** 10.1210/jendso/bvaf115

**Published:** 2025-06-28

**Authors:** Shengjie Ying, Tracy Heung, Bernice E Morrow, Bhooma Thiruvahindrapuram, Ryan K C Yuen, Anne S Bassett

**Affiliations:** Schulich School of Medicine and Dentistry, Western University, London, ON N6A 5C1, Canada; Clinical Genetics Research Program, Centre for Addiction and Mental Health, Toronto, ON M5T 1R8, Canada; The Dalglish Family 22q Clinic, University Health Network, Toronto, ON M5G 2C4, Canada; Clinical Genetics Research Program, Centre for Addiction and Mental Health, Toronto, ON M5T 1R8, Canada; The Dalglish Family 22q Clinic, University Health Network, Toronto, ON M5G 2C4, Canada; Department of Genetics, Albert Einstein College of Medicine, Bronx, NY 10461, USA; The Centre for Applied Genomics, The Hospital for Sick Children, Toronto, ON M5G 1E8, Canada; Genetics & Genome Biology, the Hospital for Sick Children, and Department of Molecular Genetics, University of Toronto, Toronto, ON M5G 1E8, Canada; Clinical Genetics Research Program, Centre for Addiction and Mental Health, Toronto, ON M5T 1R8, Canada; The Dalglish Family 22q Clinic, University Health Network, Toronto, ON M5G 2C4, Canada; Department of Psychiatry, University of Toronto, Toronto General Hospital Research Institute, and Campbell Family Mental Health Research Institute, Toronto, ON M5G 2M9, Canada

**Keywords:** polygenic risk score, 22q11.2 microdeletion, height, short stature, body mass index, copy number variation

## Abstract

**Context:**

Elevated a priori risk may enhance the likelihood that common variant effects, captured collectively in a polygenic risk score (PRS), approach clinical utility.

**Objective:**

In this study, we investigated the modifying effect of PRSs for adult height and body mass index (BMI) in individuals with elevated baseline risk for short stature (<3rd percentile height) and obesity (BMI ≥30) conferred by a 22q11.2 microdeletion.

**Methods:**

We tested height-PRS and BMI-PRS for association with their respective phenotypes in 259 adults of European ancestry with a 22q11.2 microdeletion using sequencing data and multivariable linear regression models to account for clinical/demographic variables.

**Results:**

In multivariable linear regression models, height-PRS and BMI-PRS explained 25.8% and 5.7% of the variance in their respective traits (*P* < .001 for both). When applying the height-PRS to stratify risk for short stature, 42.3% of individuals in the lowest PRS quintile had short stature (vs 5.9% in the highest PRS quintile, odds ratio = 11.46, *P* = 1.74E-05). Using logistic regression models to predict short stature in a receiver operating characteristic curve analysis, a model combining height-PRS and clinical/demographic covariates achieved an area under the curve of 0.78, performing significantly better than a covariate-only model.

**Conclusion:**

The results demonstrate that adult height and BMI can be influenced by the effects of genome-wide common variants in the presence of a rare variant conferring elevated a priori risk. Height-PRS may help refine growth expectations in individuals with 22q11.2 microdeletion.

A key challenge for rare variants is that, despite large associated risks, there is usually incomplete penetrance, leaving uncertainty about outcomes for the affected patient [1-3]. Recent studies across a broad range of diseases have suggested that the variable expression of high-impact rare variants can in part be attributable to the common variant background, captured in a polygenic risk score (PRS) [2, 4-10]. While demographic variables are usually accounted for in PRS analyses among individuals with rare variants, clinical and additional genetic variables are not.

Adult height and body mass index (BMI) are 2 clinically relevant traits that are strongly influenced by polygenic background [11, 12]. Among rare variants influencing these traits, we previously demonstrated that typical 22q11.2 microdeletions are associated with an approximately 7-fold increased risk for short stature (<3rd percentile height) [13] and 2-fold increased risk for obesity (BMI ≥30) [14], in comparison to population norms. The 1.5-2.5 Mb microdeletion that defines 22q11.2 deletion syndrome (22q11.2DS) [15] is among the most common of the rare copy number variations (CNVs) with an estimated live birth prevalence of 1 in 2148 [16]. While associated congenital, neurodevelopmental and psychiatric conditions are well-recognized [15], more recent evidence has emerged demonstrating an elevated risk for metabolic and endocrinological conditions in adulthood [14, 17-19].

The variable expression of conditions associated with the 22q11.2 microdeletion provides the opportunity to investigate how common variant background can shape the expression of a rare variant, and how such interplay may hold clinical relevance [20]. Previous studies have found that genome-wide rare variants and common variant-derived PRSs can modify the risk for expression of schizophrenia [6, 21, 22], intellectual disability [7], and hypertriglyceridemia [9], in the context of an elevated baseline risk conferred by the 22q11.2 microdeletion.

In the current study, we hypothesized that PRSs derived from general population studies may influence height and BMI in the presence of a 22q11.2 microdeletion. Using a deeply phenotyped cohort of 259 adults with a typical 22q11.2 microdeletion (Table 1, Fig. 1), we tested the association of published PRSs [12, 24] for height and BMI, adjusting for potential effects of other phenotypic variables and of 22q11.2 microdeletion extent, on height and BMI. We subsequently assessed the performance of the height-PRS for stratifying risk for short stature in 22q11.2DS by benchmarking against a clinical covariate-only model.

**Figure 1.**
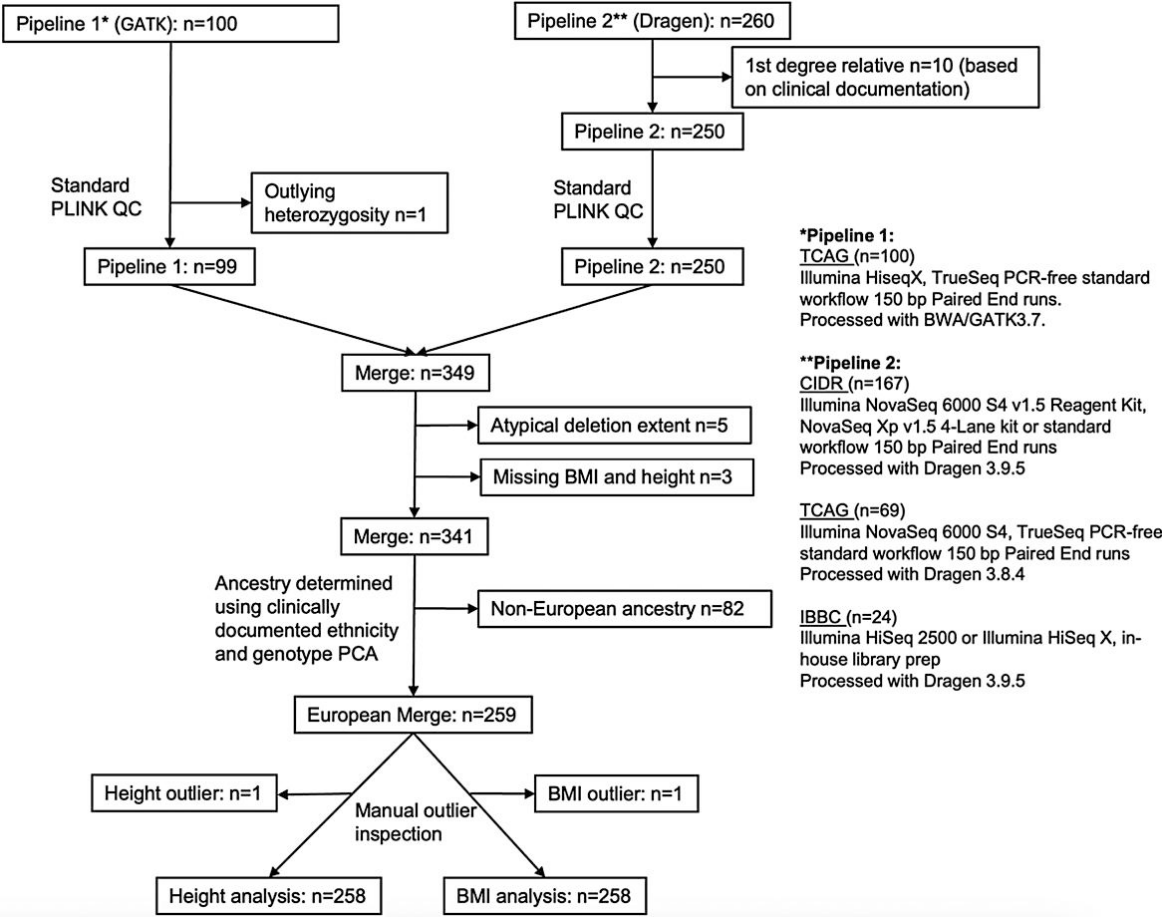
Overview of the study design. Each *n* refers to the number of individuals with a 22q11.2 microdeletion age ≥17 years. The column on the right lists the facility each set of samples was sequenced at and a summary of the sequencing platform and variant calling method. See Supplementary Figure 2 [23] for individuals identified as outliers based on height or BMI.

Abbreviations: CIDR, Center for Inherited Disease Research (Johns Hopkins University, Baltimore, USA); IBBC, International Brain and Behaviour Consortium (HudsonAlpha Genome Sequencing Center, Huntsville, USA); PCA, principal component analysis; TCAG, The Centre for Applied Genomics (Toronto, Canada).

**Table 1.** Height, BMI, and other phenotypic and genetic variables in individuals of European ancestry with a 22q11.2 microdeletion

	Total sample*^a,b^*			Males*^b^*			Females*^a^*			*P* value*^c^*
***Continuous variables***	n	Mean	SD	n	Mean	SD	n	Mean	SD	
Height (cm)*^a^*	258	162.7	9.5	119	169.3	7.2	139	157.0	7.4	**2.20E-16**
BMI (kg/m^2^)*^a^*^,*b*^	258	29.8	7.9	118	28.8	6.9	140	30.8	8.5	.**0387**
Age (years)	259	33.5	12.0	119	33.2	12.1	140	33.7	12.0	.7037

*Categorical variables*	Total sample (n = 259)*^a,b^*			Total males (n = 119)*^b^*			Total females (n = 140)*^a^*			*P* value*^c^*
Individuals with	n	%		n	%		n	%		
Short stature*^a^*^,*d*^	50	19.4		22	18.5		28	20.1		.7548
Obesity*^b,e^*	118	45.7		43	36.4		75	53.6		**8.31E-03**
LCR22A–D deletion extent*^f^*	236	91.1		112	94.1		124	88.6		.1306
Congenital heart disease*^g^*	35	13.5		17	14.3		18	12.9		.8556
Intellectual disability*^h^*	19	7.3		11	9.2		8	5.7		.3414
Psychotic illness*^i^*	94	36.3		49	41.2		45	32.1		.1541

*P* values reaching statistical significance (*P* < .05) are bolded.

Abbreviation: BMI, body mass index.

^a^One female (128.0 cm) was excluded from height analyses due to being an outlier in the height distribution (Supplementary Figure 3) [23]. This individual had a BMI of 20.5 kg/m^2^ and was not an outlier in the BMI distribution.

^b^One male (63.6 kg/m^2^) was excluded from BMI and obesity analyses due to being an outlier in the BMI distribution (Supplementary Figure 3) [23].

^c^Statistical analyses comparing males and females; *t* test for continuous variables; Fisher’s exact test for categorical values. All tests were 2-tailed.

^d^Defined as less than the third percentile for height, by sex, at age 18 years, based on World Health Organization growth curves (cutoffs: 163 cm for males, 151 cm for females).

^e^Defined as BMI ≥ 30.

^f^Those without an LCR22A–D deletion had either a LCR22A–B or LCR22A–C deletion.

^g^Defined as individuals with major (moderate-severe) congenital heart disease.

^h^Defined as individuals with moderate-severe intellectual disability.

^i^Defined as individuals with schizophrenia or schizoaffective disorder.

## Methods

### 22q11.2DS Cohort and Phenotypic and Demographic Variables

The study cohort consisted of individuals aged ≥17 years with a typical 22q11.2 microdeletion (ie, the common low copy repeat (LCR)22A–LCR22D extent or proximal nested LCR22A–LCR22B or LCR22A–LCR22C microdeletions extents) ascertained from a specialized adult 22q11.2DS clinic in Toronto, Canada. Prior to filtering and applying exclusion criteria, there were 360 individuals; 259 individuals met criteria for inclusion in primary genetic analyses (Table 1, Fig. 1). The presence of a typical 22q11.2 microdeletion was identified through standard clinical laboratory methods [18, 19] and breakpoints were confirmed using genome sequencing data.

To be included in the study, participants had to have at least one of either an adult height or BMI (height and weight) measurement obtained from clinical examination. For individuals with multiple height and weight measurements, the most recently available measurement was used, along with the corresponding age at measurement. All but 4 individuals in this adult cohort were aged 18 years or older. There were 4 individuals in the primary analyses for whom the most recently available height and weight measures available were taken at age 17 years. None of these 4 individuals had short stature. Additionally, we included other phenotypic or demographic variables that may affect height and/or BMI based on their inclusion in the phenotype-only studies of these traits [13, 14]. These were sex, age, moderate-severe congenital heart disease (CHD), intellectual disability (ID), psychotic illness, and clinically documented ancestry that was verified using genetic principal component analysis (Supplementary Figure 1) [23]. As before, we defined “psychotic illness” as individuals diagnosed with schizophrenia or schizoaffective disorder [9].

For details on genome sequencing methods and variant annotation, quality control of common variants, and principal component analysis for ancestry assignment, see Supplementary Methods [25].

### Polygenic Risk Score Regression Analyses

We used previously published gold-standard PRSs for height [12] and BMI [24]. Genotype positions and effect sizes were retrieved from the PGS Catalog [26] (height: PGS002804, BMI: PGS000027). After performing standard common variant quality control, 1 004 205 variants were used in the height-PRS (91.4% of the 1 099 005 variants in PGS002804) and 2 062 833 variants were used in the BMI-PRS (98.2% of the 2 100 302 variants in PGS000027). The PRS for each individual in the cohort was calculated using PRSice-2 [27]. Both height and BMI PRSs were derived from entirely European cohorts, and upon testing these PRSs within the European and non-European subsets of the cohort in this study, we observed dramatically diminished effect sizes in the non-European subset (Supplementary Figure 2) [23]. We therefore opted to perform our primary analyses restricted to individuals of European ancestry.

We tested for associations between the respective PRS and the height or BMI measures using linear regression (lm() function in R) in 1) a univariable model and 2) a multivariable model that adjusted for phenotypic/demographic variables, 22q11.2 deletion extent (LCR22A–D vs LCR22A–B/LCR22A–C), sequencing platform/batch (categorical variable with 4 levels: TCAG HiSeqX vs TCAG Novaseq6000 vs CIDR NovaSeq6000 vs IBBC HiSeq2500 or HiSeqX), and the first 4 principal components (PCs) of ancestry:

1. BMI or sex-standardized height ∼ corresponding PRS
2. BMI or sex-standardized height ∼ corresponding PRS + sex^a,b^ + age + deletion extent^a^ + CHD^a^ + ID^a^ + psychotic illness^a^ + sequencing platform/batch^a^ + PC1 + PC2 + PC3 + PC4
^a^Categorical variable
^b^Only included in the regression model for BMI

To adjust for the large height difference between sexes, height was represented in the regression analyses as the difference from the individual's height to the mean height of the individual's sex (ie, “sex-adjusted height”), a method previously used in the study that generated the height-PRS [12]. Sex was therefore not included as a covariate in the multivariable regression model for height. Categorical variables were treated as factors (as.factor() in R) and all continuous variables were standardized using the scale() function in R to produce beta coefficients for the regression analyses. For the sequencing platform/batch variable, CIDR NovaSeq6000 was used as the reference level in the regression model (and is thus not shown in Table 2). The increase in height or BMI per SD increase in PRS was obtained from the “estimate” coefficient ± standard error from the lm() function in R, where only the PRS values were standardized and the raw values for sex-adjusted height (cm) or BMI (kg/m^2^) were used. The variance in height or BMI that explained the multivariable model was quantified by the multiple R^2^ metric in R. The variance explained by the PRS variable alone in the multivariable model was represented by the difference in multiple R^2^ (ΔR^2^) between the full multivariable model and the multivariable model without the PRS variable. An interaction between sex and PRS for height and BMI was tested using a sex*BMI interaction term in linear regression models.

**Table 2.** Linear regression analyses testing polygenic risk score (PRS) as a predictor of height or BMI, using a univariable model and using a multivariable model adjusting for clinical/demographic, 22q11.2 microdeletion extent, sequencing platform/batch, and ancestry variables

	Height (n = 258)*^a^*			BMI (n = 258)		
	beta	std error	*P*	beta	std error	*P*
***Univariable model***						
PRS	.3492	0.0570	**3.27E-09**	.1900	0.0601	**1.76E-03**
***Multivariable model***						
PRS	.5444	0.0557	**2.00E-16**	.2429	0.0605	**8.01E-05**
Female sex*^a^*	—	—	−	.2787	0.1189	**0**.**0199**
Age	−.1071	0.0572	.0623	.1342	0.0633	.**0351**
LCR22A–D deletion extent*^b^*	−.5260	0.1871	.**0053**	−.2895	0.2113	.1720
Congenital heart disease	−.5096	0.1605	.**0017**	−.1507	0.1758	.3920
Intellectual disability	−.3971	0.2012	.**0495**	.0166	0.2227	.9406
Psychotic illness	−.0336	0.1199	.7798	.2050	0.1329	.1243
IBBC_HiSeq2500/X*^c^*	.0633	0.2918	.8284	.2919	0.3247	.3696
TCAG_HiSeqX*^c^*	.1352	0.1144	.2384	.1613	0.1277	.2076
TCAG_NovaSeq6000*^c^*	.3597	0.2084	.0857	−.1263	0.2267	.5781
Ancestry PC1	−.1681	0.0640	.**0092**	.0348	0.0694	.6159
Ancestry PC2	−.0817	0.0715	.2543	.1029	0.0752	.1724
Ancestry PC3	.0462	0.0656	.4820	−.0337	0.0724	.6417
Ancestry PC4	−.1332	0.0709	.0614	−.0560	0.0805	.4875
	**R^2^**	***P***		**R^2^**	***P***	
Model	.3386	**4.27E-16**		.1468	**3.05E-04**	
	**ΔR^2^**			**ΔR^2^**		
PRS variable	.2583			.0565		

Beta coefficients represent the SD change in the dependent variable (height or BMI) per 1 SD increase for continuous independent variables (PRS, age, and ancestry PC1-4), or when compared to the reference category for categorical independent variables (sex, deletion extent, intellectual disability, and sequencing platform/batch^c^). ΔR^2^ is an estimate of the variance explained by the PRS variable alone within a multivariable model (see Methods). *P* values reaching statistical significance (*P* < .05) are bolded.

Abbreviations: BMI, body mass index; PC, principal component; PRS, polygenic risk score; std, standard.

^a^To adjust for the large height difference between sexes (Table 1), height was represented in the regression model as the difference (cm) between the individual's height and the mean height of the individual's sex. Therefore, sex was not included in the multivariable regression model for height [12].

^b^Those who did not have the most common LCR22A–D 22q11.2 microdeletion were categorized as having a proximal nested (ie, LCR22A–­B/LCR22A–C) 22q11.2 microdeletion.

^c^To adjust for sequencing platform/batch, 1 categorical variable with 4 levels (IBBC_HiSeq2500/X, TCAG_HiSeqX, TCAG_NovaSeq6000, and CIDR_NovaSeq6000) was added to the linear regression model. CIDR_NovaSeq6000 was used as the reference category and is thus not shown.

### Risk Stratification for Short Stature Using Polygenic Risk Score

To assess the capacity of the height-PRS to stratify risk for short stature, we first stratified our cohort by quintiles of height-PRS. Short stature was defined as less than the third percentile height, stratified by sex [28]. Corresponding sex-specific cutoffs for height were determined using the third percentile growth curve at age 19 from the World Health Organization (https://www.dietitians.ca/Advocacy/Interprofessional-Collaborations-(1)/WHO-Growth-Charts/WHO-Growth-Charts-Set-2). This corresponds to a height cutoff of less than 163 cm for males and 151 cm for females. A Fisher's exact test was used to compare the proportion of individuals with short stature in the lowest vs highest quintiles of height-PRS.

Furthermore, we constructed receiver operator characteristic (ROC) curves using logistic regression models predicting short stature to calculate sensitivity and specificity of these models without the use of arbitrary cutoffs. We compared 3 logistic regression models:

1. Covariate only: short stature* ∼ sex* + age + deletion extent* + CHD* + ID* + psychotic illness*
2. PRS only: short stature* ∼ PRS
3. PRS + covariates: short stature* ∼ height-PRS + sex* + age + deletion extent* + CHD* + ID* + psychotic illness*
  *binary variable

Receiver operating characteristic (ROC) curves were created using the R package “pROC.” The difference between the area under the curve (AUC) of 2 ROC curves was compared using Delong's test for 2 correlated ROC curves and the optimal sensitivity and specificity of each ROC curve was determined using Youden's J statistic.

All statistical analyses were performed using R version 4.0.3. Statistical significance was defined as *P* < .05. All tests performed were 2-tailed. *P* values were not adjusted for multiple testing.

## Results

### Cohort Description of Individuals With a 22q11.2 Microdeletion

Table 1 summarizes the clinical, demographic, and 22q11.2 microdeletion extent features of 259 individuals (median age = 30.9 years, interquartile range = 23-41 years) of European ancestry with height and/or BMI data, along with genome sequencing data that passed quality control and other exclusion criteria (Fig. 1, Supplementary Figure 1) [23]. Similar to previous phenotypic studies of overlapping cohorts, 19.4% had short stature (median height: 169.0 cm in males, 157.5 cm in females), and 45.7% had obesity (median BMI = 29.0 kg/m^2^), representing large increases over the expected population prevalence for short stature of 3% [13] and an age-matched Canadian general population prevalence of obesity of ∼25% [14]. The only sex differences in the 22q11.2 microdeletion cohort studied related to obesity, with females having significantly greater BMI and proportion with obesity than males, and the expected shorter height in females than males (Table 1).

### Polygenic Risk Score and Other Predictors of Height and BMI

We first assessed whether PRSs for height and BMI developed in large general population cohorts [12, 24] would be associated with their corresponding traits in individuals with a 22q11.2 microdeletion. The results showed that both PRSs were significant predictors of their respective traits, with each SD increase in PRS corresponding to a 3.49 ± 0.40 cm (beta = 0.4804, *P* = 2.66E-16) increase in sex-adjusted height and a 1.54 ± 0.49 kg/m^2^ (beta = 0.1900, *P* = .0018) increase in BMI, in univariable linear regression models (Table 2).

The PRSs for both sex-adjusted height (beta = 0.5444, *P* < 2.00E-16) and BMI (beta = 0.2429, *P* = 8.01E-05) remained significant predictors of their respective traits in multivariable linear regression models that adjusted for sex (covariate used only in the BMI model), age, 22q11.2 deletion extent (LCR22A–D vs LCR22A–B/A–C), CHD, intellectual disability, psychotic illness, sequencing platform/batch, and the first 4 principal components of ancestry (Table 2). Other significant predictors (all *P* < .05) of shorter sex-adjusted height included longer (LCR22A–D) microdeletion extent, CHD, and intellectual disability. The first PC of ancestry was also significantly associated with height. Other significant predictors of higher BMI included female sex and older age (Table 2). The multivariable models explained 33.9% and 14.7% of the variance (R^2^) in height and BMI, respectively, with the PRS variables alone (ΔR^2^) explaining 25.8% and 5.7% of the variance (Table 2). There was no interaction between sex and PRS identified for either height (*P* = .218) or BMI (*P* = .702) with both PRSs having a similar effect size within sex (Supplementary Figures 4A and 5A) [23].

### Using Height Polygenic Risk Score to Stratify Risk for Short Stature

Although the PRSs for height and BMI were both significantly associated with their respective traits in linear regression models, the effect size for the height-PRS (beta = 0.480, ΔR^2^ = 0.258) was substantially greater than for the BMI-PRS (beta = 0.190, ΔR^2^ = 0.057) (Table 2). Therefore, we decided to focus on assessing the capacity of the height-PRS to stratify risk for clinically defined adult short stature.

**Figure 2.**
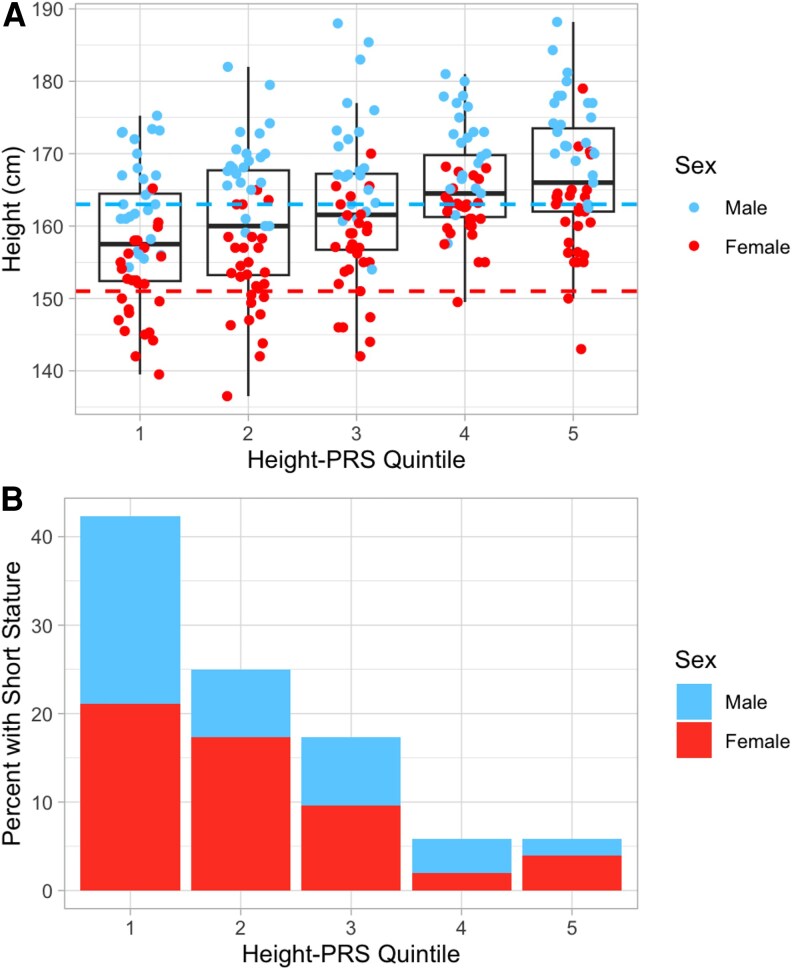
The distribution of height (A) and percent of individuals with short stature (B) per quintile of height-PRS, among adults with a 22q11.2 microdeletion (n = 258 overall; n = 119 males, n = 139 females; n = 51-52 per quintile). In panel A, the dashed blue line indicates the cutoff for short stature for males (163 cm) and the dashed red line indicates the cutoff for females (151 cm).

We first stratified our cohort by quintiles of height-PRS (Fig. 2). Across each increase in height-PRS quintile, we observed an increase in median height (Fig. 2A) and a corresponding stepwise decrease in the prevalence of short stature (Fig. 2B). Among those in the lowest height-PRS quintile, 42.3% (n = 22/52) had short stature compared to 5.9% (n = 3/51) in the highest height-PRS quintile (odds ratio = 11.46, Fisher's exact *P* = 1.74E-05).

**Figure 3.**
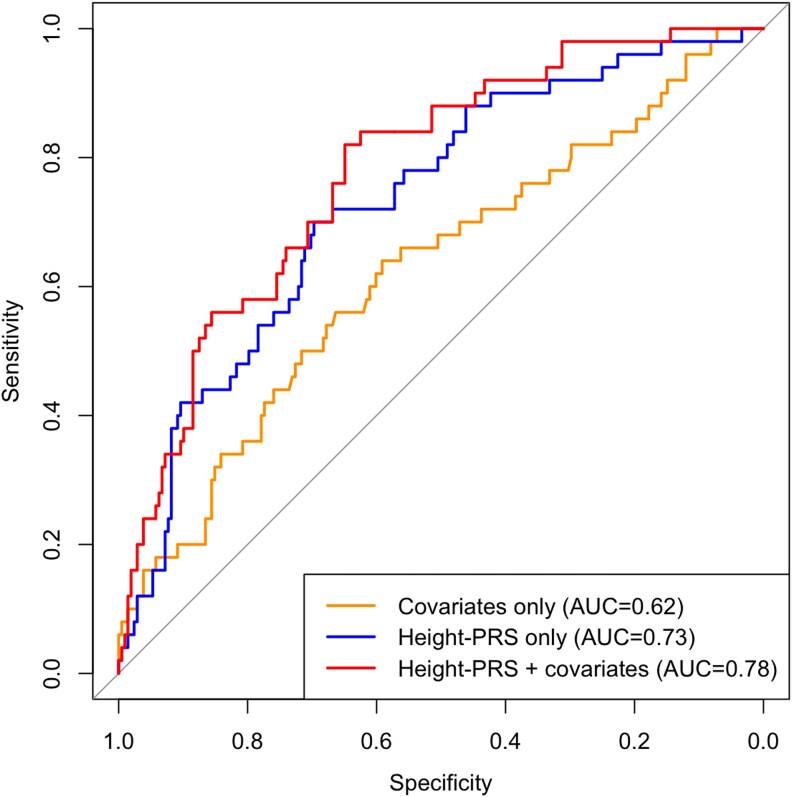
Receiver operator characteristic curves of logistic regression models predicting short stature, defined as height less than the third percentile height, by sex, at age 18 years (cutoffs: 163 cm for males, 151 cm for females), among 258 adults with a 22q11.2 microdeletion. Covariates used for the logistic regression models were sex, age, 22q11.2 microdeletion extent, moderate-severe congenital heart defect, moderate-severe intellectual disability, and psychotic illness. The area under the curve (AUC) for each curve is shown in the legend on the bottom right.

Furthermore, we constructed ROC curves using logistic regression models predicting short stature (Fig. 3, Supplementary Tables 1 and 2) [23]. The addition of height-PRS to a covariate-only model (ie, PRS + covariates model) produced a significant increase in the area under the curve (AUC) compared to the covariate-only model (AUC 0.78 vs 0.62, *P* = .0011) (Supplementary Table 1) [23]. This combined (PRS + covariates) model was the best performing model with an optimal sensitivity and specificity of 0.82 and 0.65, respectively. Also, the PRS-only model (AUC = 0.73) had a higher AUC than the covariate-only model (AUC = 0.62) (Fig. 2), although this difference did not reach statistical significance (*P* = .1124) (Supplementary Table 1) [23].

## Discussion

Adults with a 22q11.2 microdeletion have a baseline predisposition to shorter stature and obesity [13, 14]. The results of this study demonstrate that adult height and BMI are further modified by genome-wide polygenic risk, even when accounting for other clinical and demographic variables. Importantly, the addition of height-PRS significantly improved risk prediction for short stature compared to a model with only clinical/demographic covariates. Those in the lowest quintile of height-PRS had a prevalence of short stature of 42%, and were at approximately 11 times the odds of having short stature than those in the top height-PRS quintile.

PRS modification of risk for a rare variant-associated disease or condition has been demonstrated (eg, schizophrenia [6], coronary artery disease [4, 29], breast cancer [8]) and is of clinical interest. The elevated baseline risk conferred by a rare variant means that it is likely that a less extreme PRS cutoff may be required to identify individuals at high absolute risk, compared to if the same PRS were applied in a general population cohort [7]. Also, most rare variants have variable penetrance and expressivity, and PRSs may help refine the expected risk or disease severity at an individual level [4, 30]. Such proposed utility, however, requires PRSs to capture risk that is independent of risk factors that can be obtained from a clinical history. Due to the pleiotropic nature of many rare variants, other comorbid conditions associated with the rare variant may affect the trait/disease of interest and thus need to be accounted for.

Our study demonstrated the significant and independent effect of PRS, demographic variables and other 22q11.2 microdeletion-associated conditions on height and BMI (Table 2). The study design benefited from previous knowledge about these factors [13]. One such factor was the length of the 22q11.2 microdeletion. Of note, ∼85% of individuals with a 22q11.2 microdeletion have the longer LCR22A–D deletion [15]. Few phenotypes have yet been shown to be associated with this standard (vs shorter) 22q11.2 microdeletion [31]. For BMI, older age was associated with higher BMI, as expected. We also found that female sex was associated with higher BMI (Table 2, Supplementary Figure 5A) [23], consistent with general population trends in most countries [32], but that has not previously been reported in adults with 22q11.2DS [14]. Importantly, both BMI- and height-PRS remained significant predictors when adjusting for these covariates in multivariable regression models (Table 2), and height-PRS added significant predictive value when compared to a covariate-only model in an AUC analysis predicting short stature (Fig. 3).

22q11.2DS is a complex, multi-system disorder, and growth is one of many clinical features that is routinely monitored in childhood and adolescence [33]. Growth hormone therapy may be considered where there is short stature if testing indicates deficiency [33]. In the future, a height-PRS might serve as an additional useful piece of information, along with 22q11.2 deletion length, ancestry, standardized growth curves, and other clinical and demographic factors, in setting growth expectations for patients with a 22q11.2 microdeletion.

### Advantages and Limitations

22q11.2 microdeletion is an important rare genetic condition, and the study design here comprised a relatively large cohort (n = 259) with comprehensive clinical data for the study of PRS modification of height and BMI. Studies of PRS in other rare genetic conditions have used sample sizes of, for example, 44 and 50 individuals with 16p11.2 deletions and 16p11.2 duplications, respectively, for BMI-PRS [5], up to 130 individuals with a rare familial hypercholesterolemia variant for coronary artery disease PRS [4], and 20 to 90 individuals with rare monogenic forms of 5 metabolic disorders for their corresponding PRSs [2]. In contrast to these previous studies, the deep phenotyping and sample size in our study enabled identification of significant clinical and demographic predictors and a comparison of the relative predictive value of these variables with that of PRS. This provided an initial idea of what PRS may bring beyond that attained from a standard clinical history, and demonstrated the value of studying a clinical cohort ascertained using a genotype-first method for the purpose of jointly studying effects of a pathogenic rare variant, common variants, and relevant phenotypic features [34]. We note that such a study would not be possible using current large-scale biobank data due to the recognized selection bias against individuals with rare high-impact CNVs (eg, the UK BioBank has just 10 individuals with a pathogenic 22q11.2 deletion) [34, 35].

This study also has several limitations. Although the general population-derived PRSs used were associated with height and BMI in individuals with a 22q11.2 microdeletion, it is possible that the effect size of the PRS differs from when it is applied to general population samples (ie, there may be a potential interaction effect between the 22q11.2 microdeletion and PRS). For height, the variance explained by the PRS in this study (ΔR^2^) of 25.8% is lower than the most closely comparable R^2^ value of ∼40% reported in the source study [12], suggesting a possibly reduced effect size of the PRS in those with a major risk already conveyed by a 22q11.2 microdeletion. To more reliably assess for such a potential interaction, a direct comparison to a general population sample would need to be performed using matched genotyping methods, variant calling, and statistical modeling pipelines [6].

As for all current PRS studies, a critical limitation is the reduced effect size of the available PRS for non-European populations [36] (Supplementary Figure 2) [23], which unfortunately compelled us to exclude the non-European subset of our sample from the primary analyses (Fig. 1). We also note that there was no notable improvement among the non-European subset when we used the multi-ancestry height-PRS (PGS002802). Also, although the sample represents, to our knowledge, the largest cohort available of individuals with a 22q11.2 microdeletion with adult height, BMI, and genome sequencing data, our sample size for this rare disease is, by definition, small compared to studies of the general population [12, 37]. We included an extensive list of clinical/demographic covariates that may influence height and/or BMI in our linear regression models (Table 2); however, there are other potentially relevant factors that were not consistently accessible among the adult patients in this study, such as mid-parental height, childhood growth hormone treatment, immunodeficiency status, calcium status, smoking, sedentariness, and family history of obesity. Lastly, given the preliminary nature of these results, still limited predictive accuracy, and absence of any consensus on potential clinical actionability, the findings do not support clinical use of these PRSs at this time. Future studies are also needed to assess for the contribution of additional factors such as other genome-wide rare variants and lifestyle factors in 22q11.2DS.

## Conclusion

In summary, PRSs for height and BMI are associated with expression of their respective traits in individuals with a 22q11.2 microdeletion where a priori risks for short stature and obesity are elevated. The results demonstrate that genome-wide common variants can modify the major effects of a rare CNV on adult height, even while accounting for other contributory factors. While promising for height, further research will be needed before polygenic risk achieves individual-level clinical utility as a predictor of this adult outcome for this high-impact rare variant.

## Data Availability

Restrictions apply to the availability of some or all data generated or analyzed during this study to preserve patient confidentiality or because they were used under license. The corresponding author will on request detail the restrictions and any conditions under which access to some data may be provided. The datasets generated and/or analyzed during the current study, including the raw genome sequencing and clinical data, are not publicly available due to the sensitive nature of the data. The polygenic risk scores (PRSs) used in this study are listed in the Methods and are publicly available in the PGS Catalog (https://www.pgscatalog.org/). The code and calculated individual-level PRSs used for analyses are available from the corresponding author upon reasonable request.

## Abbreviations

22q11.2DS: 22q11.2 deletion syndrome
AUC: area under the curve
BMI: body mass index
CHD: congenital heart disease
CNV: copy number variation
PRS: polygenic risk score
ROC: receiver operating characteristic
